# Learning of Central Pattern Generator Coordination in Robot Drawing

**DOI:** 10.3389/fnbot.2018.00044

**Published:** 2018-07-23

**Authors:** Payam Atoofi, Fred H. Hamker, John Nassour

**Affiliations:** Artificial Intelligence, Computer Science, Chemnitz University of Technology, Chemnitz, Germany

**Keywords:** robot learning, motor coordination, coordination transfer, central pattern generator, robot drawing

## Abstract

How do robots learn to perform motor tasks in a specific condition and apply what they have learned in a new condition? This paper proposes a framework for motor coordination acquisition of a robot drawing straight lines within a part of the workspace. Then, it addresses transferring the acquired coordination into another area of the workspace while performing the same task. Motor patterns are generated by a Central Pattern Generator (CPG) model. The motor coordination for a given task is acquired by using a multi-objective optimization method that adjusts the CPGs' parameters involved in the coordination. To transfer the acquired motor coordination to the whole workspace we employed (1) a Self-Organizing Map that represents the end-effector coordination in the Cartesian space, and (2) an estimation method based on Inverse Distance Weighting that estimates the motor program parameters for each SOM neuron. After learning, the robot generalizes the acquired motor program along the SOM network. It is able therefore to draw lines from any point in the 2D workspace and with different orientations. Aside from the obvious distinctiveness of the proposed framework from those based on inverse kinematics typically leading to a point-to-point drawing, our approach also permits of transferring the motor program throughout the workspace.

## 1. Introduction

The capacity of applying previously acquired skills in a new context is referred to as transfer of learning, e.g., the potential advantage of using the experience from a previously performed motor task to learn a new motor task. The transfer may happen between different tasks or between different contexts. The transfer of motor tasks in robotics is an essential alternative to learning from scratch when facing a new task or facing a new context. However, how could robots generalize their motor program? How are motor skills represented? Lashley (1951) introduced the concept of a motor program as the sequence of movements that can be prepared in advance and executed without feedback. The idea of a motor program was later described by Keele (1968) as a structured combination of muscle commands before the movement sequence starts, which can produce a sequence of movements regardless of feedback. One of the evidences for a motor program's existence in humans is a control structure, such as Central Pattern Generators (CPG) (Morris et al., 1994; Summers and Anson, 2009). CPG neurons, located in the spinal cord of vertebrates, are responsible for locomotion (Brown, 1911; Shik et al., 1966). In vertebrates the spinal cord could generate movements without sensory feedback, but it also receives input from different areas of the brain within a hierarchical structure (Jahn et al., 2008). Motor neurons of the peripheral nervous system receive input from the central nervous system and drive muscles. In turn, muscles act in coordination to produce movements. In robotics, movements are generated through a model-based approach or are acquired by means of model-free algorithms (Bertram et al., 2006; Schaal, 2006; Saegusa et al., 2009; Ijspeert et al., 2013; Siciliano and Khatib, 2016), each of which has its own challenges and benefits. Due to the way our task is defined, the aforementioned approaches used in drawing, writing or similar arm motions shall be briefly reviewed here. Singh et al. uses a robot's inverse kinematics model for a point-to-point drawing scenario (Singh et al., 2015; Singh and Nandi, 2016). In their work, the absence of the concept of the motor program would result in the inability to transfer the produced movements into a new context. Pastor et al. (2009) provides an approach for learning motor skills based on human demonstration. The robot movement is produced by learning non-linear differential equations in task space, then a velocity-based inverse kinematics model is used to calculate the movement parameters in the joint space. The movement is generalized with respect to the goal position that is explicitly expressed in the dynamic equations. In their work the motor coordination is implicitly encoded by the inverse kinematic model, i.e. no coordination parameters are explicitly used to control the motion in the joint space. In Pastor et al. (2009) and Singh and Nandi (2016), a kinematic model is essential to produce a movement. A model-free approach however requires gathering data and extracting necessary information by a robot experiment, or by a robot demonstration, e.g., imitation learning. Tan et al. (2012) proposed a model-free algorithm for a robot to learn writing by imitation. Motor patterns are generated based on Dynamic Movement Primitives (DMPs). Since a semantic knowledge learning approach has been applied to associate a motion to a drawn pattern (numbers: 0–9), generalization is not possible in order to draw a new pattern which is not previously demonstrated. Mochizuki et al. (2013) has employed a Multiple Timescales Recurrent Neural Network (MTRNN) that generates the next action according to the current joint angle and the next end-effector position. MTRNN is first trained through body-babbling to associate the arm dynamics to the end-effector dynamics. The network is retrained afterwards to produce basic shapes (triangle, rectangle, and circle) that were demonstrated by a human. However, the motion is generated as a sequence of small movements instead of a single motor pattern in the joint space for drawing one line in the task space, which results in a less smooth drawing. Calinon et al. (2007) generalized the robot's task trajectory, acquired by demonstration, using a probabilistic method, Gaussian Mixture Regression, and a dimensionality reduction technique (PCA). A generalized joint trajectory is produced based on the expected end-effector position, joints positions, and object distance over time. Saegusa et al. (2009) developed an internal model for reaching for a robot's arm through an exploration algorithm called motor-babbling-based sensory motor learning. The coordination is expressed by a function called “confidence” that measures the reliability of state prediction and the motor command, where the high value of the confidence points to a reliable knowledge of state dynamics. The state-action association is not generalized along the workspace, instead, the robot performs an exploration phase first then an off-line learning phase.

We here present an approach of how the motor coordination is transferred to a new context when executing a motor task by a humanoid robot. Drawing has been chosen as the robot's task, however, this work can be extended to any task that involves coordination. Movements are generated by a central pattern generator model that can produce rhythmic and discrete motor patterns, Multi-Layered Multi-Pattern Central Pattern Generator (MLMP-CPG) (Nassour et al., 2014). In addition to its advantage of being supported in the biological domain, as compared to DMPs, the MLMP-CPG's multi-layer separation also provides us with the opportunity to control coordination parameters without influencing the nature of the generated pattern. Motor coordination is composed of a spatial and a temporal part within the CPG model. A NAO humanoid robot initially learns to draw lines with 8 different directions from the same starting position inside the workspace. The acquired motor coordination is then transferred using self-organizing map (SOM) to other starting positions in the workspace. The robot is finally able to reproduce any visually presented pattern by extracting the straight lines and drawing them inside the reachable workspace. Each line is drawn by a single CPG pattern generated at each joint, unlike inverse kinematics methods that perform the task by connecting a sequence of points.

The computational model MLMP-CPG is briefly introduced in section 2. Section 3 shows the acquisition of motor coordination parameters for drawing lines with 8 different directions and with different lengths performed by the robot's arm with two degrees of freedom. This has been achieved by a multi-objective optimization. Section 4 describes the proposed framework to transfer motor coordination within the reachable workspace and contains the main numerical results. The experiment on the real robot is presented in section 5. A conclusion is provided in section 6.

## 2. Movement generation

**Figure 1:**
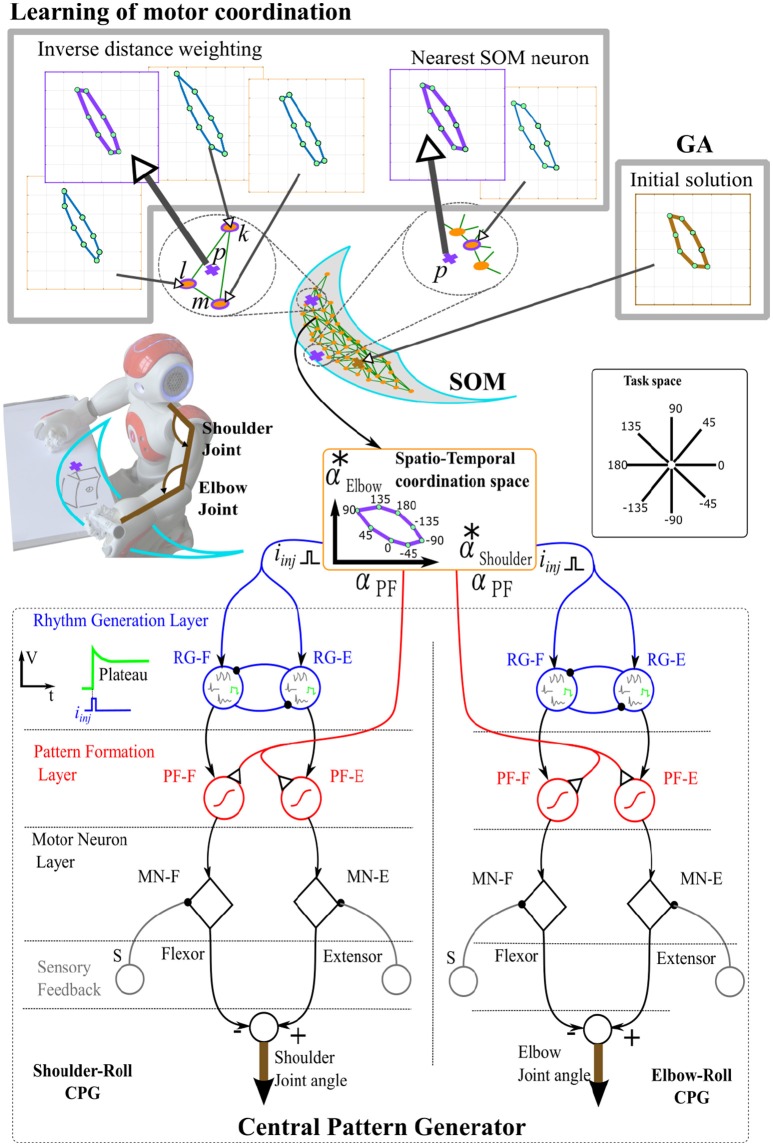
An illustration of the learning algorithm in combination with the Central Pattern Generator. Plateau patterns are selected at the Rhythm Generation layer (RG), other patterns can also be generated such as quiescent, oscillation, and almost oscillation (Rowat and Selverston, 1997). The temporal coordination is specified by the injected currents in RG neurons, while the spatial coordination is determined by the slope α_*PF*_ of the sigmoid activation function in the Pattern Formation layer (PF). A Self-Organizing Feature Map (SOFM) represents the workspace, and, furthermore, coordination parameters ($\alpha_{\mathrm{ShoulderRoll}}^{*},\alpha_{\mathrm{ElbowRoll}}^{*}$) are associated to each of its neurons, where they are used to coordinate a line drawing action from any given starting point of the workspace. The motor coordination acquisition is done by applying a multi-objective genetic algorithm, whose result is used later for the transfer algorithm in the learning of motor coordination. As shown in the learning of motor coordination block, the estimation of the coordination parameters is done either by Inverse Distance Weighting (IDW) interpolation or nearest SOM neuron.

To produce movements, the central pattern generator model (MLMP-CPG) proposed by Nassour et al. (2014) has been used in Debnath et al. (2014). This CPG model has three layers: rhythm-generation layer (RG), pattern-formation layer (PF), and motorneuron layer (MN), see Figure 1. Extensor and flexor neurons in the rhythm-generator layer are expressed by (1) to (5):

(1) $$\tau_{m}\frac{dV}{dt}=-(\mathrm{fast}(V,\sigma_{f})+q-i_{\mathrm{inj}}),$$

(2) $$\tau_{s}\frac{dq}{dt}=-q+q_{\infty }(V),$$

(3) $$\tau_{m}<\tau_{s},$$

(4) $$\mathrm{fast}(V,\sigma_{f})=V-A_{f}\tanh((\sigma_{f}/A_{f})V),$$

(5) $$q_{\infty }(V)=\sigma_{s}(V-E_{s}),$$

where *V* is the membrane potential, and represents the *RG* neuron output. *q* and *q*_∞_ are the slow current and its steady state value, respectively. *A*_*f*_ determines the width of the N shape of the current-voltage curve. τ_*m*_ and τ_*s*_ are time constants. *i*_inj_ is the injected current, and it is equal to −1 or +1 for both directions of movement (clockwise or counterclockwise). σ_*s*_ and σ_*f*_ represent the conductances for potassium and calcium currents, respectively. *E*_*s*_ is the reversal potential. With different values of cell parameters, four patterns are generated: quiescence, almost an oscillator, oscillations, and plateau. These patterns are illustrated for the schematic RG neurons in Figure 1. Since this paper addresses a line drawing task, we only use the plateau pattern. Other patterns can be used when the task space involves more complex movements. Pattern formation neurons (*PF*_*E*_, *PF*_*F*_) receive input from rhythm generation neurons. Their activation function is expressed by (6):

(6) $$PF=\frac{1}{1+e^{\alpha_{0}\cdot \alpha (\psi_{0}-w_{\text{RG}\to \text{PF}}\cdot V)}},$$

where α_0_ = 1 is the slope of the sigmoid, ψ_0_ = 0 defines the center point. α represents the descending control from the high-level controller that modulates the activation of the pattern formation neuron *PF*, which is shown in Figure 1 as α_*PF*_. *w*_RG → *PF*_ is the weight of the synaptic connection between *RG* and *PF* neurons. Each *PF* neuron projects to the corresponding motor neuron (*MN*). The activation function of each extensor and flexor motor neuron is expressed by (7):

(7) $$MN=\frac{1}{1+e^{\xi (\beta -(w_{\text{PF}\to \text{MN}}\cdot PF+w\cdot S)/2)}},$$

where, ξ = 5 and β = 0.5 are the slope and threshold of the sigmoid activation function, respectively whose values were set empirically. *w*_PF → MN_ is the weight of the synaptic connection between *PF* and *MN* neurons. *S* is the proprioceptive sensory feedback, *w* is its corresponding weight. In the current study *w* is set to 0, because no sensory feedback is considered at the motorneuron level.

Patterns generated at each joint are coordinated by descending control signals. The temporal coordination between joints occurs by the injected current *i*_inj_, i.e., the activation at the rhythm generation layer of each CPG. The spatial coordination is done by specifying the slope of the sigmoid activation function, through α, in the pattern formation layer, see Figure 1.

## 3. Motor coordination acquisition for drawing

To obtain a proper motor coordination while learning to perform a task, an error signal needs to be introduced and minimized through a trial and error process. In case of learning to draw lines from different starting positions and with different attributes, the error could be defined as a vector of two error signals, e.g., errors in the angle and length of the line of the performed trial. Therefore, the optimization process of drawing a line from a given starting position is considered as a multi-objective task, where the error signals of the angle and length are the two objectives to be minimized. The problem consists in finding the coordination parameters that satisfy both objectives. However, the objectives might be conflicting and a trade-off becomes substantial to satisfy one objective against another. As a pattern generator, the CPG model presented in section 2 has been used. The manipulation of all the CPG parameters of each layer would result in a large action space, yet keeping certain parameters constant will allow us to cover only those actions reasonable for the defined task. Parameters at the rhythm generation layer that select the nature of the pattern were initiated to generate a plateau pattern at each joint (Shoulder-Roll and Elbow-Roll). Coordination parameters between robot's joints are represented by a set of CPG parameters (α_PF_ and *i*_inj_) for each joint, representing spatial- and temporal coordination, respectively. A coordinated movement corresponds to the proper selection of parameters α_PF_ in the pattern-formation layer (spatial coordination) and *i*_inj_ in rhythm-generation layer (temporal coordination). In order to draw a line in task-space with two degrees of freedom in joint space, both joints need to start their motion simultaneously. However, they can be in phase or in opposite phase. Therefore, only the direction of the injected current would be of concern. Thus, both coordination parameters (α_PF_ and *i*_inj_) are merged and represented as a single parameter *signed-alpha*, α^*^:

(8) $$\begin{array}{l} |\alpha^{*}|=\alpha_{\text{PF}},\\ \mathrm{sgn}(\alpha^{*})=\mathrm{sgn}(i_{\mathrm{inj}}). \end{array}$$

**Figure 2:**
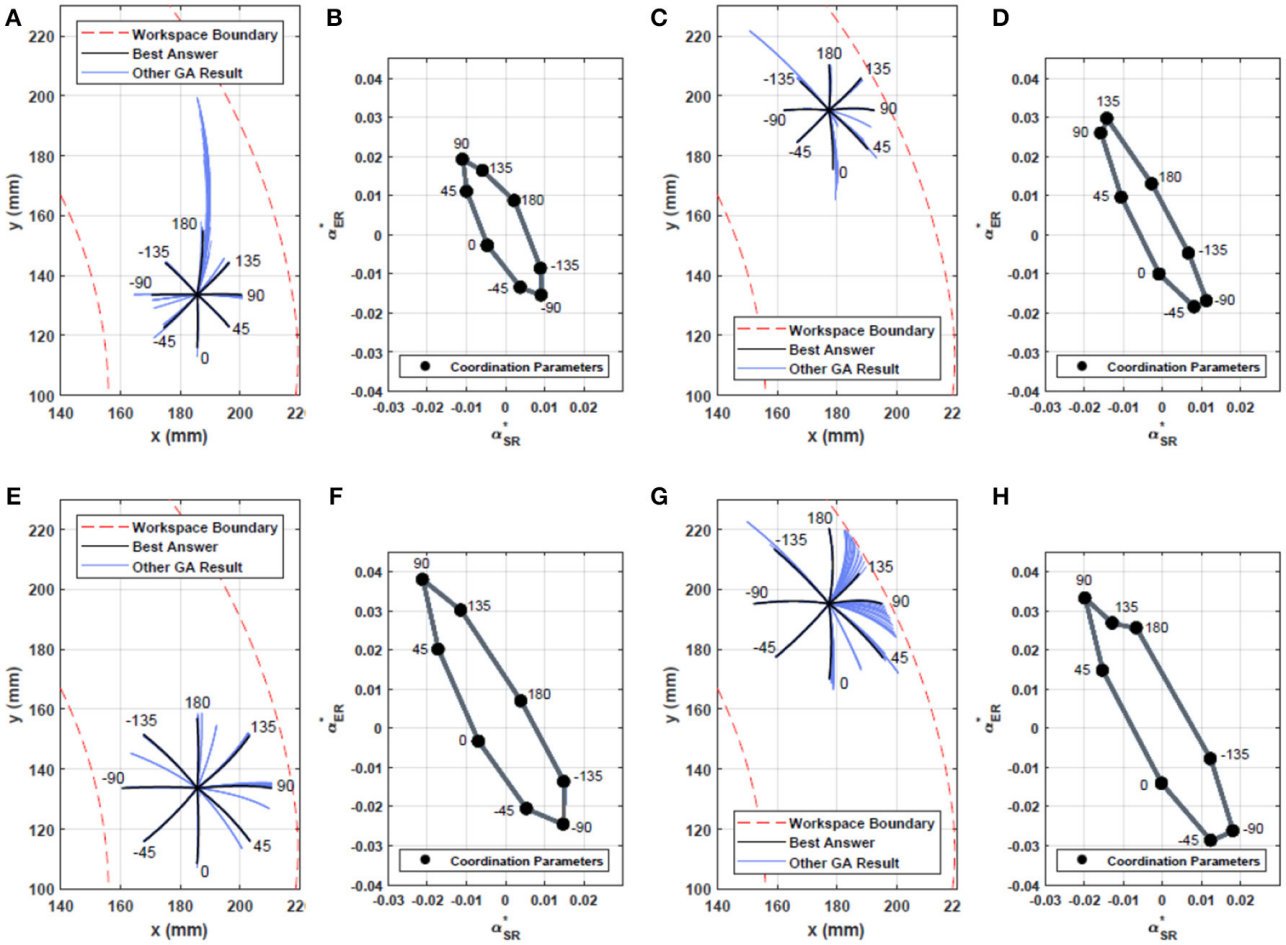
Motor coordination to draw lines with eight desired angles (0, 45, 90, 135, 180, −135, −90, −45) using a multi-objective Genetic Algorithm. **(A–C)** show the resulting drawing from the optimization at two different arm initial positions with a line length of 15*mm*. **(B,D)** show the coordination parameters of the black lines in **(A,C)** that resulted from the optimization process. Each point represents parameters (α and *i*_inj_) of a spatio-temporal coordination for a drawn line. The horizontal axis represents the coordination parameters for the Shoulder Roll (SR), the vertical axis for the Elbow Roll (ER). **(E–H)** represent the resulting optimization for a line length of 25*mm*. Although there exists no linear mapping from a point on Cartesian workspace and its coordination parameters to a different point (and its coordination parameters), **(A–D)**, there is however a linear relation between coordination parameters of lines drawn from one point with similar angle but with different lengths, **(B,F,D,H)**. In **(G)**, due to the starting position being close to the boundary of the workspace, it can be seen that for the line with angle 135° and length of 25*mm* our method settled for the coordination parameters with a length smaller than the desired, hence **(H)** not being the scaled version of **(D)**.

**Figure 3:**
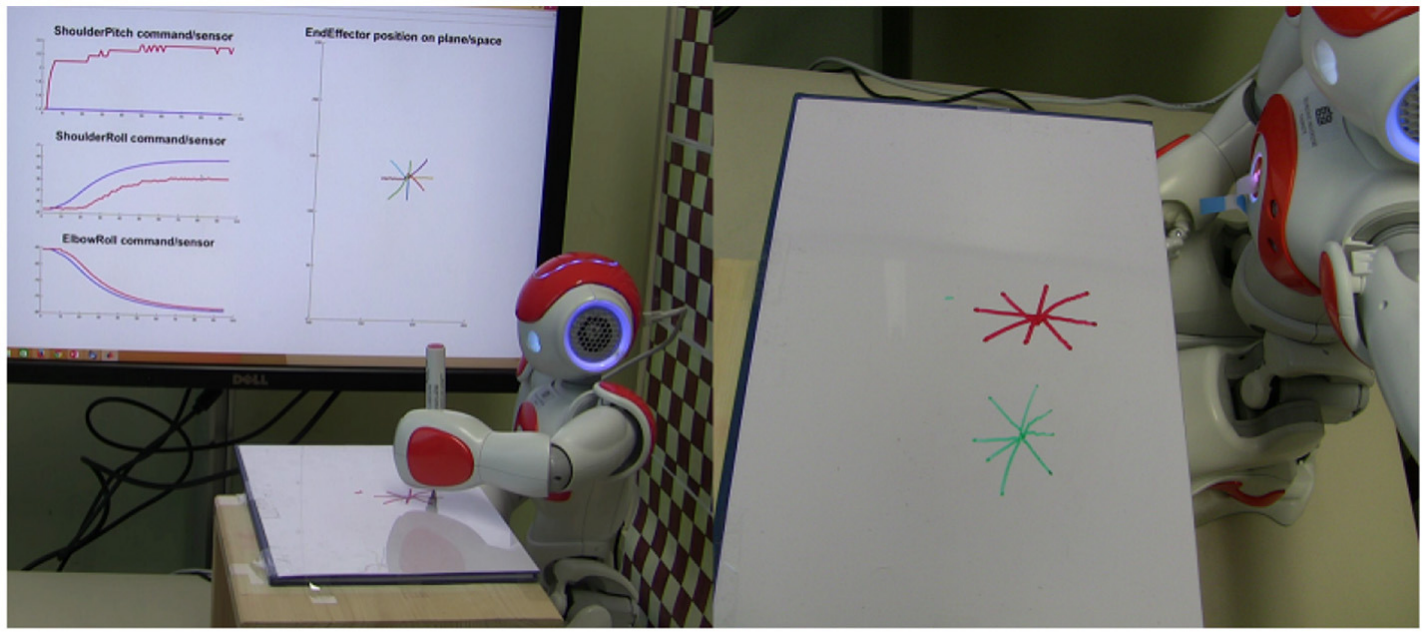
NAO humanoid robot drawing lines for the obtained motor coordination (motor coordination parameters from Figures 2F,H). The robot draws eight desired lines at each of the two initial positions.

The method of choice should be suitable to optimize a function with two objectives. Another criterion of choosing an optimization method is knowing whether the derivative information of the function is available. Based on the above mentioned criteria a multi-objective genetic algorithm was used to solve the optimization problem (Haupt and Haupt, 2004; Konak et al., 2006). The goal is to find appropriate coordination parameters (α^*^) in each CPG for drawing lines. The optimization toolbox in MATLAB provides us with the multi-objective genetic algorithm, *gamultiobj*, which was opted and used with its default parameters, i.e., crossover function, crossover fraction, distance measure of individuals, mutation function, size of the population, etc. The number of variables is 2 for $\alpha_{\text{SR}}^{*}$ and $\alpha_{\text{ER}}^{*}$. The objectives (*f*_1_, and *f*_2_) are given in (9):

(9) $$f_{1}=\left|\frac{\theta -\theta_{d}}{180}\right|,f_{2}=\left|\frac{l-l_{d}}{100}\right|,$$

where θ and *l* are the angle and the length of a drawn line, respectively. θ_*d*_ and *l*_*d*_ are the desired values. When a joint reaches a mechanical limit before the end of the action, the trajectory resulted from such motion would not be a straight or close to a straight line. Therefore, the individual in the population that represents the action will be given significantly high values for *f*_1_ and *f*_2_. Due to the large number of iterations, the optimization process was run in simulation. We used a kinematic model of NAO robot's left arm. Figure 2 illustrates the acquired motor coordination in drawing lines with eight desired angles (0, 45, 90, 135, 180, −135, −90, −45°) and two desired lengths (1.5, 2.5*cm*), from two different starting positions in the workspace. Figures 2A,C,E,G show the optimized drawing patterns in task space. Final solutions are in black. Figures 2B,D,F,H show the corresponding motor coordination parameters of those drawn in black. Figure 3 shows the robot drawing 8 lines from the two initial positions previously presented in Figures 2F,H. Results presented in Figure 2 show that the coordination parameters for drawing lines with same lengths from different initial positions cannot be scaled linearly along the workspace.

## 4. Motor coordination transfer

How can the coordination parameters be transferred from one starting position to another without learning from scratch? How can one obtain a representation for the motor coordination of the line drawing task over the workspace? We address the problem of transfer of motor coordination by employing an interpolation algorithm that uses previously acquired coordination parameters for an action in several arm configurations to estimate the coordination parameters for the same action in a new not-previously-experienced arm configuration. We also use a SOM to represent the coordination parameters over the workspace.

### 4.1. Representing motor coordination over the workspace

The initial arm position influences the motor program of drawing. Moreover, if two arm configurations are close to each other in joint space, their corresponding motor programs for a specific action, including coordination parameters, will also be close to each other. Hence, a Self-Organizing Map (SOM) was employed to represent the Cartesian workspace, while preserving the neighborhood of similar parameters in the coordination space, i.e., α^*^. Therefore, each neuron in the map represents the end-effector position which corresponds to an arm configuration and the associated motor coordination parameters. The SOM is an unsupervised learning technique for obtaining a neighborhood topology based on competitive learning (Kohonen, 1982). The update formula for a neuron *v* with its associated weight *w*_*v*_(*s*) is expressed in (10):

(10) $$w_{v}(s+1)=w_{v}(s)+h(u,v,s).\gamma (s).(n(t)-w_{v}(s)),$$

where *s* is the step index, *t* is the index of training sample, *u* is the index of the winner for the input vector *n*(*t*), γ(*s*) is a learning coefficient, which decreases monotonically, *h* is the Gaussian neighborhood function which provides the distance of the neuron *u* from the neuron *v* in step *s*. Each SOM neuron, which represents an area in the workspace, is associated with a motor coordination vector of *signed-alpha*s, which was obtained by a weighted mean over all of the coordination parameters (*signed-alpha* vectors) of the data in a cluster represented by that neuron. The arm starting positions were selected randomly in the workspace with 1*cm* distance from the boundaries to allow the robot to draw lines in all directions (with at least 1*cm* length).

Figure 1 illustrates the transfer algorithm. An initial coordination for a given point in the workspace is provided by the multi-objective genetic algorithm. Then, the estimation of the coordination parameters is based on either Inverse Distance Weighting (IDW) interpolation or nearest SOM neuron. The estimation algorithm provides a set of proper α^*^ vectors for the desired action at a newly visited starting position *p* (shown with cross), where the α^*^ parameters for different actions are shown in coordination-space (the 8 green circles representing the coordination for drawing the 8 desired lines, with horizontal axis for the Shoulder Roll and vertical axis for the Elbow Roll). If the starting position is surrounded by previously trained neurons, with coordination parameters associated to them, an interpolation using IDW will be used, whereas for other starting positions, the coordination parameters from the nearest trained SOM neuron will be transferred.

### 4.2. Interpolation by inverse distance weighting

We applied an interpolation based on Inverse Distance Weighting (IDW) (Shepard, 1968). The idea is to find the set of spatiotemporal parameters that produces the least error in drawing the desired lines from a new starting point in the workspace. For a random arm starting position *p*, three surrounding neurons (if existed) *k*, *l*, and *m* are involved in the interpolation process (Figure 1) to estimate the coordination parameters for the point *p* (distance-based IDW):

(11) $$\alpha_{p}{}^{*}=\frac{\sum_{i\;=\;k,l,m}w_{i}\cdot \alpha_{i}{}^{*}}{\sum_{i\;=\;k,l,m}w_{i}},w_{i}=\frac{1}{d(p,i)},$$

where *d*(*p, i*) is the Euclidean distance between the point *p* and a neuron *i*.

We used “point in triangle test.” One of the triangle vertices is the closest SOM neuron to the point “*p*.” The other two neurons are selected from the neighbors of the first vertex, but only the two which would encompass the point “*p*” as well as form a triangle with the smallest area. If the point *p* is not surrounded by three neurons (Figure 1), only the closest neuron will be considered to estimate the coordination parameters at that point. The resulting estimated coordination vector at point *p* is used as the 1st trial on the robot/simulation. Therefore, eight lines with possibly different direction other than the desired ones will be drawn based on the estimation for eight desired lines. The angular errors of the drawn lines are used in another IDW process, angle-based, that optimizes the coordination parameters needed to draw the 8 desired lines at point *p*:

(12) $$\alpha^{*}(\theta )=\frac{\sum_{i\;=\;b,a}w_{i}(\theta )\alpha_{i}{}^{*}}{\sum_{i\;=\;b,a}w_{i}(\theta )},w_{i}(\theta )=\frac{1}{\left|e(\theta ,\theta_{i})\right|},$$

where *b* and *a* are the indices of the drawn lines with the closest angles (the line before and the line after) to the desired angle with positive and negative errors. *e* is the difference between the desired angle and the current angle *i*, whose absolute value would be used as the criterion of success. The angle-based IDW iteration of trials will continue until the 8 drawn lines become close to the desired lines with an acceptable error of 1° for each line, where the optimization process terminates. The resulting coordination parameters are therefore associated to the point *p*, which will ultimately change the associated coordination parameters of a neuron representing a cluster, in which point *p* lies. It has been pointed out that the starting values for the optimization has been obtained from the very first estimation (early estimation) of coordination parameters and their corresponding initial errors resulted from the action. Hence, the initial error ought to be ever reducing throughout the training of the SOM network.

### 4.3. Results

**Figure 4:**
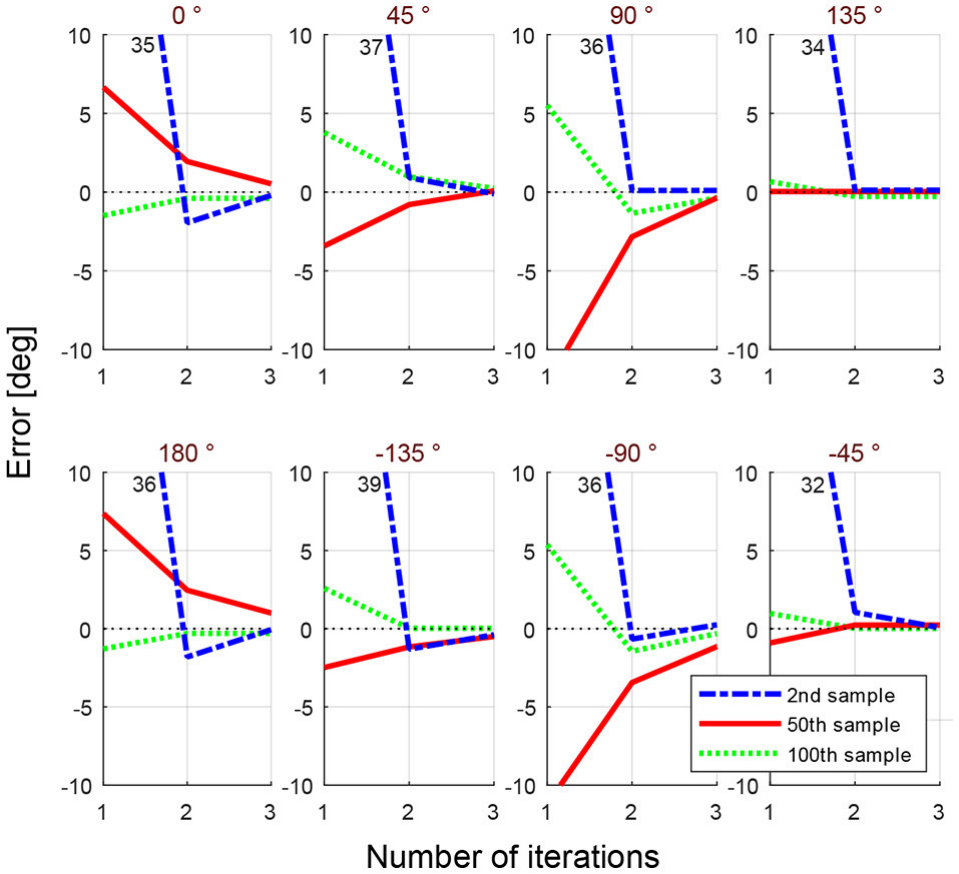
Error in eight desired angles for three selected samples during the training. Each sample corresponds to a point in the workspace. Blue lines show the optimization for the 2nd random starting position, red lines for the 50th, and green lines for the 100th randomly selected sample. Through iteration, using (12), the error is reduced to reach an acceptable margin.

**Figure 5:**
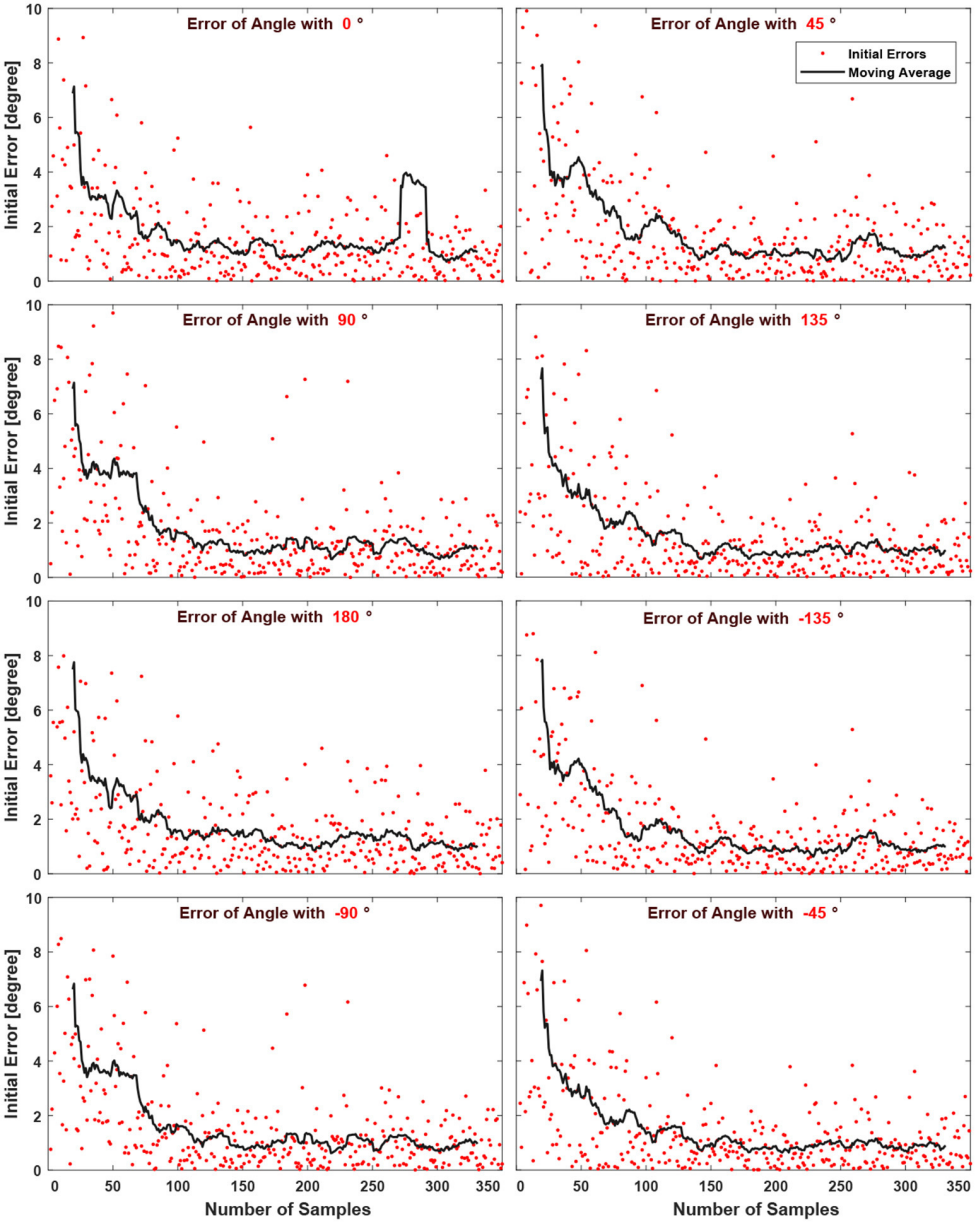
The absolute error of early estimation (initial error) decreases with increasing samples for each desired line angle. Each sample represents a randomly selected arm configuration in the 2D workspace. The moving average, black line, in each figure further illustrates this effect.

**Figure 6:**
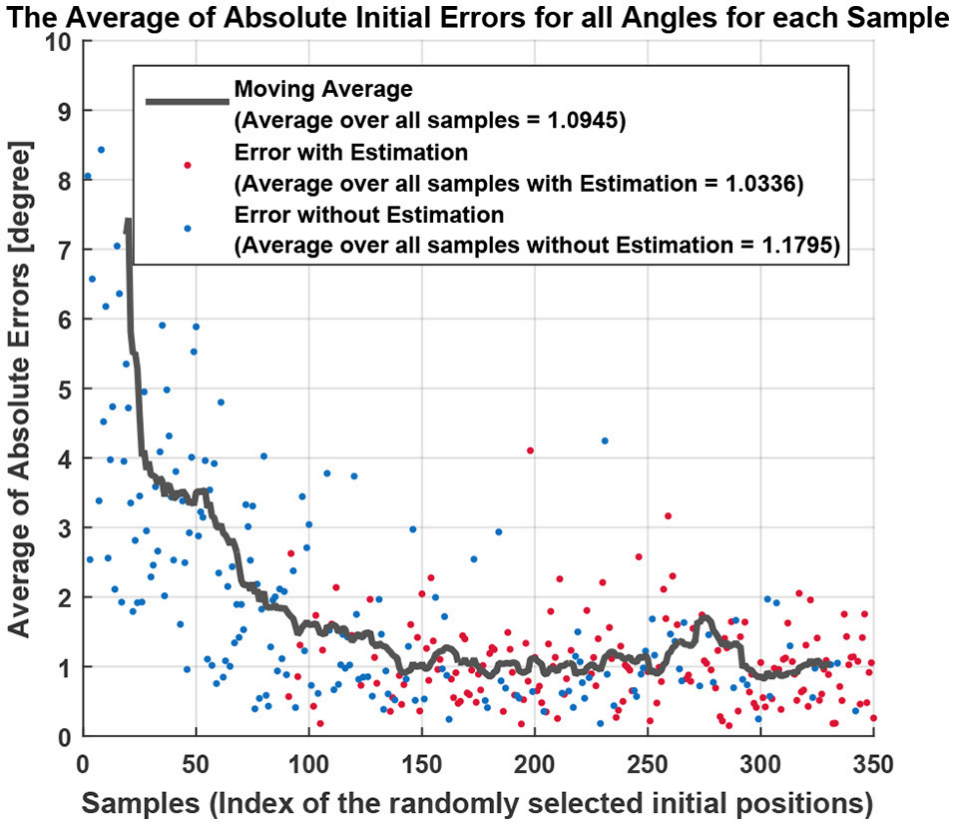
The average of absolute initial errors of all desired angles are shown for all trials. The initial errors were shown separately for each angle before in Figure 5. Depending on the the starting point (sample) the coordination parameters were provided either by the closest trained neuron to the sample, in blue, or by an estimation using IDW (11) among the surrounding neurons, in red.

All the methods explained in this section so far require at least one initial position with its coordination parameters available, that is why the coordination parameters of the very first initial position (1st sample) is given by a multi-objective GA. Having a point in the workspace, whose coordination parameters to draw the eight desired lines are found by GA, allows the second initial position (2nd sample), which is chosen randomly on the workspace, to use the IDW for the optimization of its coordination parameters. Figure 4 shows the initial error values for three randomly generated samples (points) in the workspace. Lines with eight desired angles have been optimized using IDW to reduce the error resulted from the early estimated coordination parameters. It can be seen that the estimated coordination parameters for drawing a line with 0° resulted in an initial error of 35° in drawing that line from a starting position of the end effector at the 2nd randomly selected point in the workspace, while the error is about 6° for the 50th randomly selected point in the workspace. The significant error reduction in early estimation over samples is due to the training of the SOM network, enabling its neurons to represent clusters of samples of previously experienced points in the workspace. To better capture the effect of the amount of samples on the error of early estimation (or initial error, the error resulted from the first estimated coordination parameters before the optimization process begins), Figure 5 shows the absolute error of the estimation algorithm over the samples. At the start, the error was high for most of the drawn lines. The decreasing trend of the errors along the number of samples is shown by the moving average, the black lines. After enough number of samples, the absolute errors are minimized (the average of the absolute errors of all the eight drawn lines becomes about 1° in Figure 6), which is a direct result of the SOM neurons scattering in the workspace, where each neuron represents a cluster of samples, and holds coordination parameters for drawing eight lines with 8 desired angles. If a sample has a considerably high error, caused by an inaccurate interpolation, it will influence the moving average noticeably, as it can be seen in Figure 5 for the error of angle with 0°. Figure 6 shows the average of total error of eight angles drawn at each trial, where the moving average, black line, shows the descending behavior in the error. Blue dots show the average error for eight desired lines of those trials with starting points in Cartesian workspace that are not surrounded by trained SOM neurons, therefore, selecting the initial coordination parameters from the nearest SOM neuron. Red dots show the average of the error (for eight lines) of the trials with starting points located in an area surrounded by trained SOM neurons, where the coordination paramters are estimated via IDW of the surrounding SOM neurons, using (11). At the early stages of learning, the coordination parameters for drawing lines from a newly selected point in the workspace is often initialized only by the coordination parameters of the nearest trained neuron, since not many of the neurons are trained. Whereas, it is initialized by IDW of the surrounding neurons at late stages of learning.

## 5. Robot experiment

To show the effectiveness of the proposed generalization technique over a two dimensional workspace, we introduced drawing patterns consisting of simple shapes that can be reconstructed by only straight lines. First, the robot extracts the straight lines' features, e.g., the position of a starting point, the angle, and the length of each line. Second, the robot runs an interpolation method based on the straight line's starting point in the workspace, by the selection of the neurons in the SOM network surrounding that point. A distance-based IDW (Equation 11) will be employed to estimate the coordination parameters α^*^ for drawing eight lines based on the coordination parameters of the surrounding neurons. If the angle of the presented line was not in the list of eight desired angles (0, 45, 90, 135, 180, −135, −90, −45°), an angle-based IDW (Equation 12) will be run to find an estimate of coordination parameters. To find the motor coordination parameters of a line with a length other than the learned line length (1.5*cm*), a coefficient λ will be used that linearly maps α^*^ for the learned line length into new coordination parameters α^*^ for a new length. λ is a varying coefficient related to the ratio of the length of the desired line (*l*_*n*_) to the length of the line whose coordination parameters are available (*l*_*a*_), $\lambda \propto \frac{l_{n}}{l_{a}}$.

### 5.1. Image processing

**Figure 7:**
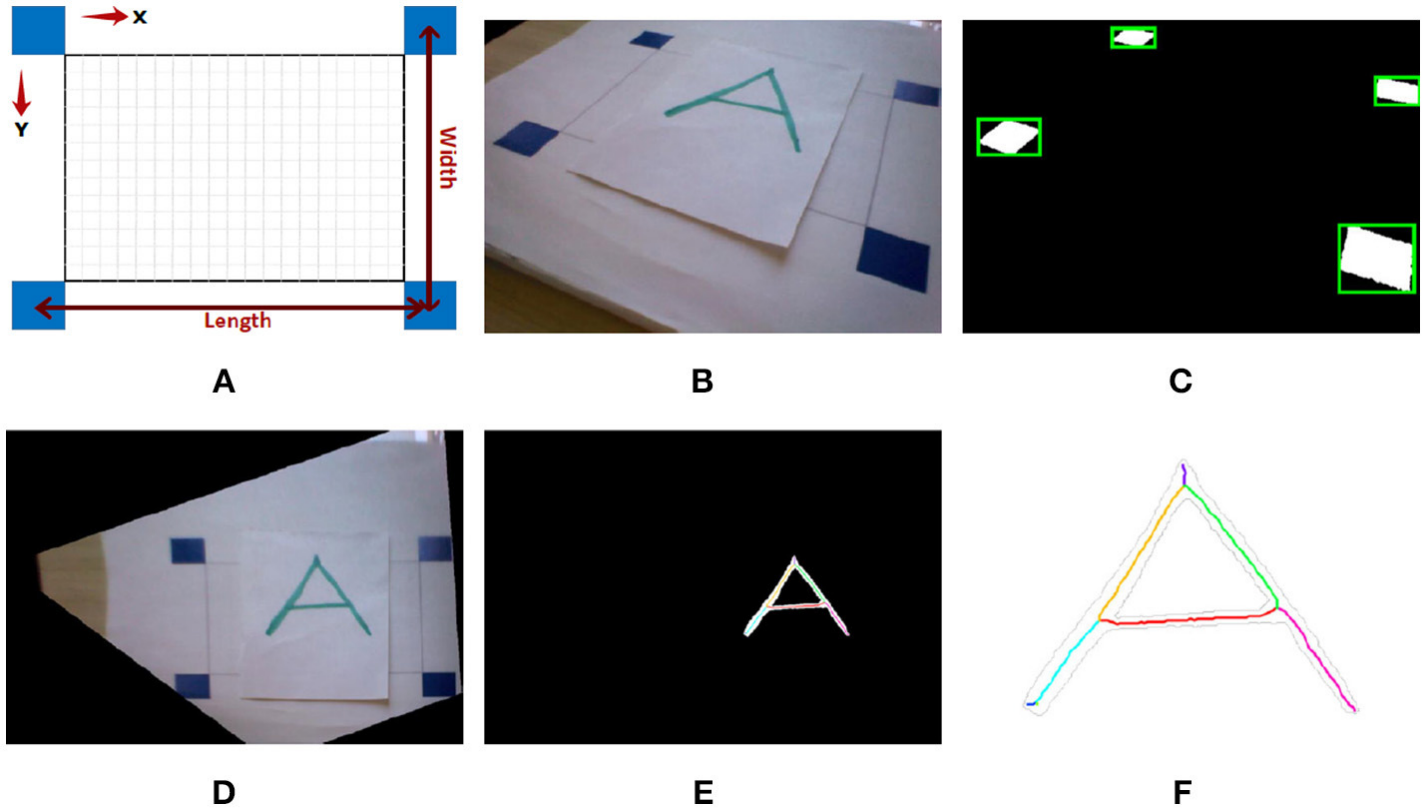
Result of image processing, transformation and line segmentation. **(A)** Depicts the reference source which is printed on a paper and is placed in front of the camera, where all the 4 blue rectangles are inside the camera view. **(B)** Is the caputred image by NAO's bottom camera. **(C)** Shows the result of image processing to detect the 4 blue corners necessary for the image transformation (Homography). Having the coordinates of the 4 blue corners creating an irregular quadrilateral, the captured image can be projected to a plane where these corners create a rectangle, whose result is shown in **(D)**. **(E,F)** are the results of image processing and line segmentation after transformation.

A line segmentation and an image transformation method has been employed to extract image features. After getting an image from the robot's bottom camera (Figure 7B), the four blue squares in the image will be detected by filtering out the range of colors not used in the squares, then converting the image to a binary image, and finally measuring the properties of different regions of the binary image (Figure 7C). Since the images captured by the camera are within a workspace, where the normal of the plane of the workspace is *not* perpendicular to the camera view, the angles, and the lengths of the lines in the image are not the real angles and lengths with respect to the robot's world frame (Figure 7A). Therefore, we performed an image homography transformation (Corke, 2017). Considering this is a planar projective transformation (*homography*), it will be a linear transformation and can be represented as follows:

(13) $$\left(\begin{array}{l} wx^{\prime }\\ wy^{\prime }\\ w \end{array}\right)=\left[\begin{array}{ccc} h_{11} & h_{12} & h_{13}\\ h_{21} & h_{22} & h_{23}\\ h_{31} & h_{32} & h_{33} \end{array}\right]\left(\begin{array}{l} x\\ y\\ 1 \end{array}\right),$$

where *x, y* and *x*′, *y*′ are the coordinates in the camera view and its projection to another plane, respectively. The four points that are needed to solve the system of elements of the projection matrix has been chosen as the four blue rectangular corners of a rectangle placed on the workspace, see Figure 7A. Figures 7B–D show the captured image, the image processing required for the homography transformation and its result.

To avoid taking the varying width of the pattern into account, and to focus on the shape of the pattern rather than the thickness (sketch drawing of lines) a morphological operation, “a thinning algorithm” (Lam et al., 1992), has been used, see Figures 7E,F. In the segmentation phase, segments are found by extracting junctions based on Kovesi (2000). Straight lines are extracted afterwards from each segment by following the changes in slope throughout the segment.

### 5.2. Line length calculation

The line length (*l*_*line*_) is obtained by calculating the line projections ($l_{x}^{realworld}$, $l_{y}^{realworld}$) on the *x*- and *y*-axis. We calculate the relation of the length of the line with respect to the width/length of the rectangle in the image space. Given the actual width and length of the rectangle in the real world, we obtain the length of the line by (14):

(14) $$\begin{array}{l} l_{x}^{realworld}=\frac{l_{x}^{im}}{l_{rec}^{im}}\times l_{rec}^{realworld},\\ l_{y}^{realworld}=\frac{l_{y}^{im}}{w_{rec}^{im}}\times w_{rec}^{realworld},\\ l_{line}=\sqrt{{\left(l_{y}^{realworld}\right)}^{2}+{\left(l_{x}^{realworld}\right)}^{2}}, \end{array}$$

where, $l_{x}^{im}$ and $l_{y}^{im}$ are the projections of the line on the *x*- and *y*-axis, respectively in image space, calculated in pixel. $l_{rec}^{im}$ and $w_{rec}^{im}$ are the length and the width of the rectangle in image space, calculated in pixel. $l_{rec}^{realworld}$ and $w_{rec}^{realworld}$ are the length and width of the rectangle in the Cartesian space.

### 5.3. Mapping from image space to joint-space

For a given point within the reachable workspace in the image space, we need to find joint angles that move the arm to that point. This mapping between the image space and the joint space is essential to determine the initial position of the arm for drawing a line from a given position in the image space. To solve the mapping there are two main approaches, the first is by using the kinematic model of the robot (model-based), while the second is by learning the mapping, e.g., by a neural network (model-free). We adopted the latter where no kinematic model is required. To associate image space with joint space we collect a training set by moving the left arm within the 2D workspace, where a marker with a fixed distance from the end-effector is attached to the arm. The value of Shoulder Roll and Elbow Roll joints were measured and associated to the marker position in the image. To generalize the association for the non-visited points in the workspace we fed the training set to a two-input/two-output multilayer perceptron neural network (MLP) that has one small hidden layer (six neurons). The network's training function was selected as Bayesian Regularization algorithm to maximize the log likelihood or to minimize the Mean Squared Error (MSE). The transfer function for hidden layers were selected as hyperbolic tangent “*tanh*” and the transfer function for output layer was linear function [*f*(*x*) = *x*]. After the network has learned the association, each starting position in the image space will be mapped with an acceptable error into an arm configuration that moves the end-effector to that starting point.

### 5.4. Drawing multiple lines

**Figure 8:**
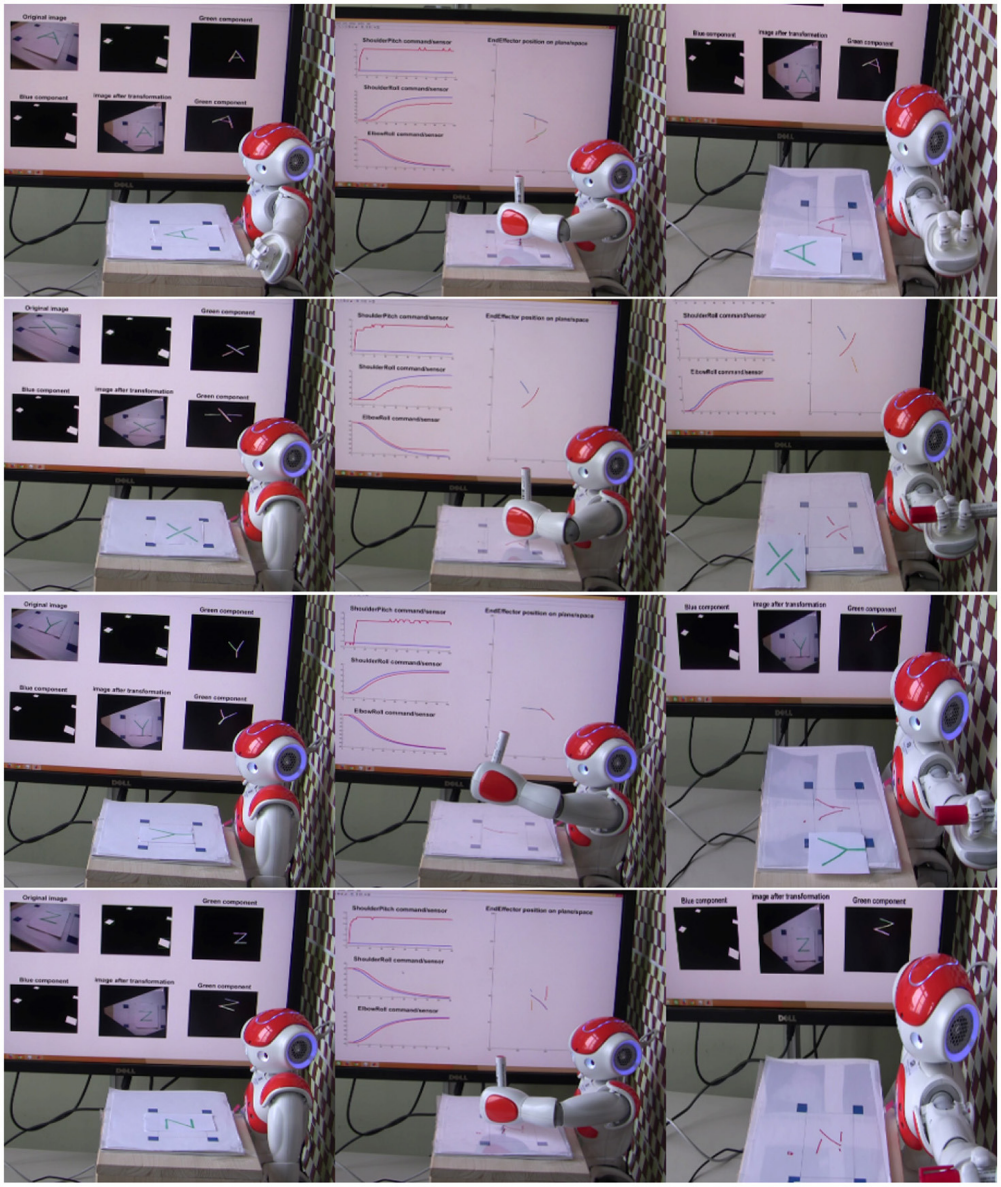
NAO draws letters A, X, Y, and Z that consist of only straight lines. A video is available on: https://www.tu-chemnitz.de/informatik/KI/edu/robotik/videos/TMCRDT.mp4.

Figure 8 shows the robot drawing different presented shapes consisting of only straight lines. The white board is manually positioned in front of the robot in such a way that the four blue markers are inside the reachable workspace so that the robot is able to reach any point within the rectangle. It is worth mentioning that the workspace was fixed in front of the robot in a way that its normal is parallel to the *z*-axis of base frame in torso. A routine as a preliminary to the start of the action would ensure that the hand loosen its grip for the pen to slide toward the white board until it reaches the board before holding it firmly again. Letters “A,” “X,” “Y,” and “Z” are presented to the robot. First, the robot extracts the lines in each letter (without any semantic learning). Each line will be labeled with a starting position, a length, and an angle. Coordination parameters to draw a line with a given angle and length at that starting position are estimated based on the trained SOM. With the help of the MLP by having a mapping from image space to joint space, the arm's initial position (joint angles for shoulder roll and elbow roll) is calculated. At this initial position, then, the robot performs an action using the CPG coordinated patterns to draw a straight line. Afterwards, the arm is moved to a new starting position to draw another line.

## 6. Conclusion

We proposed a framework to learn the motor coordination of the CPG patterns with respect to the arm's initial position in the workspace. The spatio-temporal coordination is represented by two parameters in the central pattern generator model, which are generalized throughout the workspace by employing an inverse distance weighting algorithm that interpolates the coordination space for previously visited initial arm positions. The resulting motor program was used to draw lines from different starting points in the workspace. The IDW method optimizes only one objective, angle of a line, which requires at least two starting samples of lines with the same length. In this paper, parameters that define the nature of the generated pattern (plateau, quiescent, oscillator, almost an oscillator) are not involved in the transfer process. The modularity of the motor program simplifies the transfer problem by only emphasizing the role of the involved modules such as the spatio-temporal coordination modules in the generalization of a given task over the workspace. This simplification requires the modules defining the nature of the action to remain unchanged. Unlike previously proposed algorithms for robot drawing, each line in the task space is represented by only one action in the joint space, which explains the smoothness of the obtained drawing. The proposed drawing scenario is supported by studies on scribbling stage of drawing during the human development (Gardner, 1982; Quaglia et al., 2015), which also inspired the animation studies in computer graphics (Noris et al., 2012). In this study, plateau patterns are used at the joint space to draw straight lines at the task space. However, an extension to draw more complex patterns will be possible by involving other CPG patterns such as quiescent, oscillatory, etc. to allow drawing shapes not only consisting of straight lines.

## Author contributions

All authors listed have made a substantial, direct and intellectual contribution to the work, and approved it for publication.

### Conflict of interest statement

The authors declare that the research was conducted in the absence of any commercial or financial relationships that could be construed as a potential conflict of interest.
